# Surveillance of genomic diversity and antimicrobial resistance in enterotoxigenic Escherichia coli in England, 2015–2023

**DOI:** 10.1099/jmm.0.002081

**Published:** 2025-10-03

**Authors:** Delia-Gabriela Grigoruta, Ching-Ying J. Poh, Ella V. Rodwell, Adam Crewdson, Satheesh Nair, Claire Jenkins

**Affiliations:** 1 Gastrointestinal Bacteria Reference Unit, UK Health Security Agency, London, UK; 2 University of Coventry, Coventry, UK; 3 National Institute for Health and Care Research Health Protection Research Unit in Gastrointestinal Infections, University of East Anglia, Norwich, UK; 4 Gastrointestinal Infections & Food Safety (One Health), UK Health Security Agency, London, UK

**Keywords:** antimicrobial resistance, enterotoxigenic *Escherichia coli*, molecular typing, surveillance, traveller's diarrhoea

## Abstract

**Introduction.** Enterotoxigenic *Escherichia coli* (ETEC) are one of the leading causes of gastrointestinal infections globally, primarily affecting children in low- and middle-income countries and travellers to endemic regions.

**Gap Statement.** The surveillance of diarrhoeagenic *E. coli* in England focuses on Shiga toxin-producing *E. coli*, and the true clinical and public health burden of ETEC is unknown. This gap extends globally, as many countries, particularly those in endemic regions, lack the infrastructure, diagnostic tools and healthcare facilities to resource surveillance programmes for ETEC.

**Aim.** The aim of this study was to utilize available data to describe the epidemiology, genomic diversity and antimicrobial resistance (AMR) of ETEC in England.

**Methodology.** A total of 587 isolates of ETEC cultured from faecal specimens referred to the Gastrointestinal Bacteria Reference Unit at the UK Health Security Agency for further testing, from 2015 to 2023, were sequenced to determine sequence type (ST), serotype, virulence and AMR profiles, and integrated with epidemiological data obtained from referral forms.

**Results.** Overall, the number of ETEC notifications increased annually, with a 35.5-fold increase from 2015 to 2023. There were more female cases (51.7%) than males (48.3%), with the highest proportion of cases belonging to the 50–59 age group (18.6%). Nearly half of the cases (49.5%) were travel-associated, with Egypt, Pakistan, India, Turkey and Mexico being the top travel destinations. At least 139 STs and 132 serotypes were identified, with the most common ST-serotype profiles being ST4 O6:H16 (*n*=74) and ST182 O169:H41 (*n*=66). Genome-derived AMR data revealed widespread resistance to fluoroquinolones and *β*-lactams, including third-generation cephalosporins, and over 40% of isolates (*n*=239/587) were resistant to three or more classes of antimicrobials.

**Conclusion.** We observed an increase in notifications of multidrug-resistant ETEC over the last decade, mainly associated with travellers’ diarrhoea. Nationwide expansion of PCR-based diagnostics for ETEC, alongside strengthening collaboration with public health agencies and genomic data sharing at a local, national and international level, is critical for strengthening surveillance and accurately assessing the true burden of ETEC locally and on a global scale.

## Data Summary

All FASTQ files and assemblies were submitted to the NCBI. Illumina FASTQ and Nanopore FASTQ accessions can be found under BioProject: PRJNA315192 (Table S4, available in the online Supplementary Material).

## Introduction

Gastrointestinal (GI) infections are a public health burden worldwide, causing nearly two billion cases with mortality rates exceeding one million annually [1]. Children under the age of five are vulnerable, especially in low- and middle-income countries (LMICs), accounting for approximately half of the total deaths associated with GI infections [2]. In England, nearly 17 million GI infections and over one million general practitioner consultations occur yearly, placing a financial burden on the National Health Service at a cost of over £60 million [3]. GI infections also cause significant socio-economic hardship; high rates of school and work absenteeism, decreased productivity and declining consumer confidence linked to high-risk food items all have an economic impact [4,5].

Diarrhoeagenic *Escherichia coli* (DEC) are among the most common pathogens responsible for GI infections worldwide [6]. DEC can be classified into five different pathotypes, including Shiga toxin-producing *E. coli* (STEC), enteropathogenic *E. coli*, enteroinvasive *E. coli*, enteroaggregative *E. coli* and enterotoxigenic *E. coli* (ETEC). Globally, ETEC is the most prevalent DEC pathotype and the leading cause of traveller’s diarrhoea (TD), accounting for up to 60% of total TD cases globally [7]. Primarily affecting children under the age of five in LMICs, ETEC contributes to high child mortality rates, reaching up to 500,000 deaths annually [8,9]. ETEC have been detected in the animal reservoir, specifically in ruminants and pigs, and persist in various environmental settings, including food, soil and water [10]. Transmission to humans occurs via contaminated food and water and/or direct or indirect contact with colonized animals or their environment. Humans may also act as transient vectors, via person-to-person contact or by contaminating food or water with human faeces [11].

The clinical presentation of ETEC infection is characterized by early onset acute watery diarrhoea without blood [12]. Cholera-like symptoms, including fever, abdominal pain and vomiting, are usually common, especially in children [13,14]. After ingestion, ETEC reaches the small intestine, attaches to the gut epithelial cells via colonization factors and produces ST (STp and STh variants) and/or LT enterotoxins [15]. These enterotoxins ultimately result in water and electrolyte secretion in the intestinal lumen, leading to acute watery diarrhoea [16].

Although ETEC infection is mostly self-limiting, with oral or intravenous rehydration therapies used as the recommended treatment [17], antimicrobial therapies are sometimes recommended in severe cases or for high-risk individuals, including young children and immunocompromised patients [18]. The overuse or misuse of antibiotics in diarrhoeal disease management has led to the emergence of antimicrobial resistance (AMR) strains, especially in LMICs, where antibiotics are commonly administered without prescription [19,20]. The World Health Organization has classified AMR ETEC as a critical public health threat [21].

The surveillance of DEC in England focuses on STEC, due to its association with severe clinical outcomes, such as haemolytic uraemic syndrome, and the true clinical and public health burden of ETEC is unknown. This gap extends globally, as many countries, particularly those in endemic regions, lack the infrastructure, diagnostic tools and healthcare facilities to resource surveillance programmes for ETEC [22,23]. We conducted a retrospective analysis of genome sequences of ETEC isolates from faecal specimens referred to the Gastrointestinal Bacteria Reference Unit (GBRU) between 2015 and 2023 to look for epidemiological trends, determine the population structure and identify virulence and AMR profiles. By integrating epidemiological data and genomic findings, this study aims to present a detailed, comprehensive analysis of the surveillance data to inform disease severity and better understand the public health impact of ETEC in England.

## Methods

### Microbiology

Clinical infection is diagnosed by detecting ETEC in faecal specimens using PCR targeting *sth*, *stp* and *lt*. Although ETEC are not included in the local hospital diagnostic microbiology testing algorithm in the UK, faecal specimens submitted to the GBRU at the UK Health Security Agency (UKHSA) for testing for STEC are concurrently tested for ETEC. Microbiological results are stored in the Gastro Data Warehouse (GDW), an in-house UKHSA database for storing and linking patient demographic and microbiological typing data. Patient information including sex, age and recent travel was collected from laboratory request forms upon submission and stored in GDW.

### DNA extraction, sequencing and data quality control

Genomic DNA from all isolates was extracted using the QIAsymphony (Qiagen). The sequence library was prepared using the Nextera XT DNA and Nextera Flex sample preparation kits (Illumina) and sequenced using the Illumina HiSeq 2500 and NextSeq 1000 platforms (100 bp paired-end reads) at UKHSA. Trimmomatic (v0.32) was used to trim sequence adapters. Quality metrics require a minimum sequencing yield of 150 Mbp and a minimum reference genome coverage of 30×. Reads which fell below a PHRED score of 30 from the leading and trailing ends, or with a read length less than 50 bp, along with their pairs, were discarded. FASTQ reads from all sequences in this study can be found at the UKHSA Pathogens BioProject at the National Center for Biotechnology Information (NCBI; BioProject number PRJNA315192).

### Sequence typing

Sequence type (ST) assignment was performed using a modified version of short-read sequence typing (SRST) using the MLST (v2.15–2.17) database described by Tewolde *et al.* [24]. The MOST software (for MLST) is available at https://github.com/phe-bioinformatics/MOST. For the isolates with a clean 7-gene MLST allelic profile, GrapeTree was employed to generate a minimum spanning tree (MSTree-V2) [25]. This was visualized in the GrapeTree platform and annotated with ST derived from SRST2. Where there was a probable ST (due to the database not holding all the allelic variants), this was checked in EnteroBase [26].

### Virulence and AMR profiling

Virulence profiling was performed via *GeneFinder* (v2.9–2.11) (https://github.com/phe-bioinformatics/gene_finder). Genes were confirmed to be present if the coverage and sequence similarity were over 85% compared to the reference gene. AMR profiling was performed, and the presence of AMR genes was determined using *GeneFinder* (https://github.com/phe-bioinformatics/gene_finder), as well as the UKHSA in-house database. AMR profiles are displayed using UpSetR (http://gehlenborglab.org/research/projects/upsetr/).

## Results

**Fig. 1.**
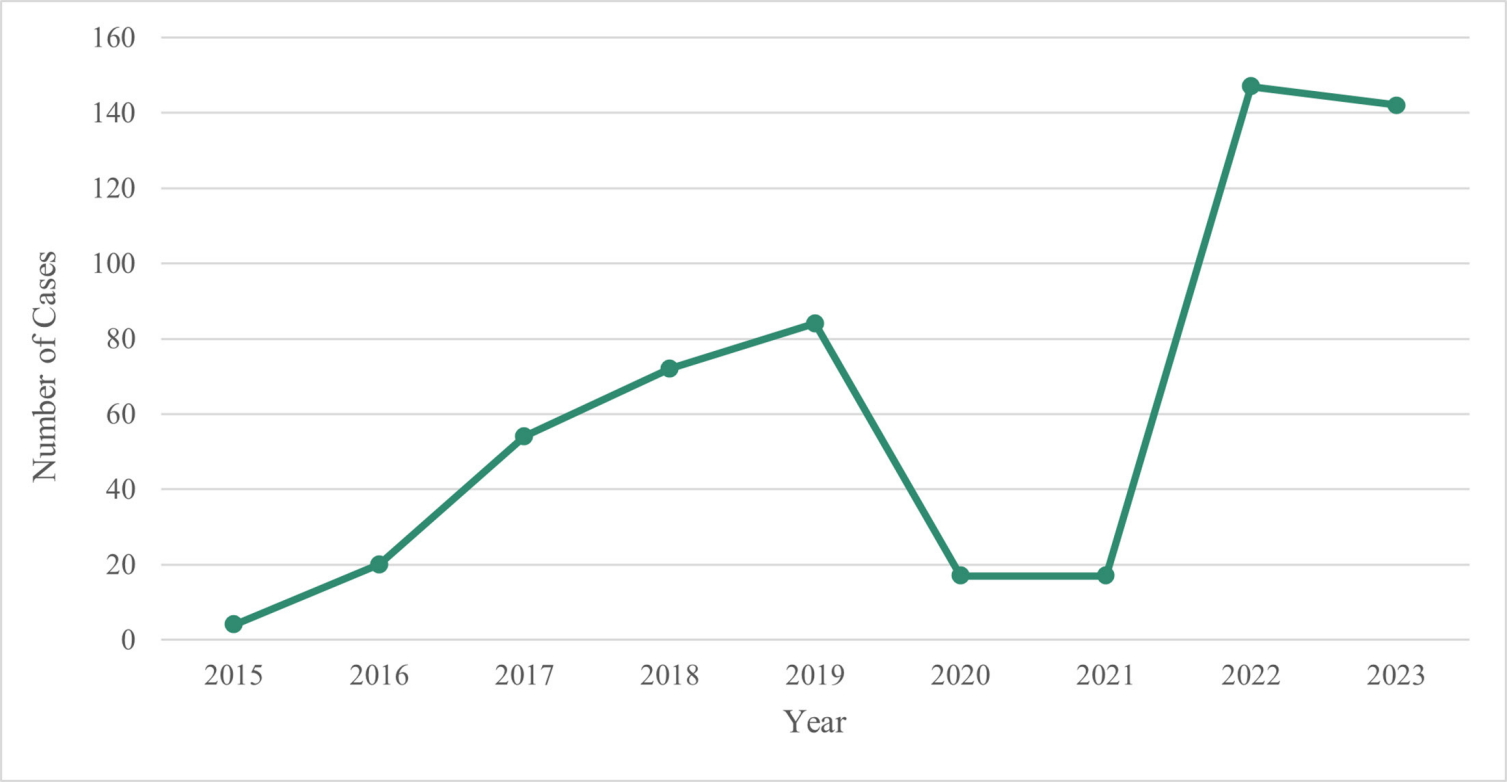
Incidence of ETEC cases submitted to GBRU between January 2015 and December 2023 (*n*=557).

The dataset comprises 587 isolates of ETEC from 557 patients submitted to the GBRU at UKHSA between January 2015 and December 2023. There were 30 patients that submitted more than one faecal specimen and had multiple isolations of ETEC. There was a year-on-year increase in notifications of ETEC, from four cases in 2015 to 142 cases in 2023, representing a 35.5-fold rise over the study period (Fig. 1). A sharp decline was observed in 2020–2021 (*n=*34), but the number of cases resumed an upward trend in 2022 (*n*=147) (Fig. 1).

**Fig. 2.**
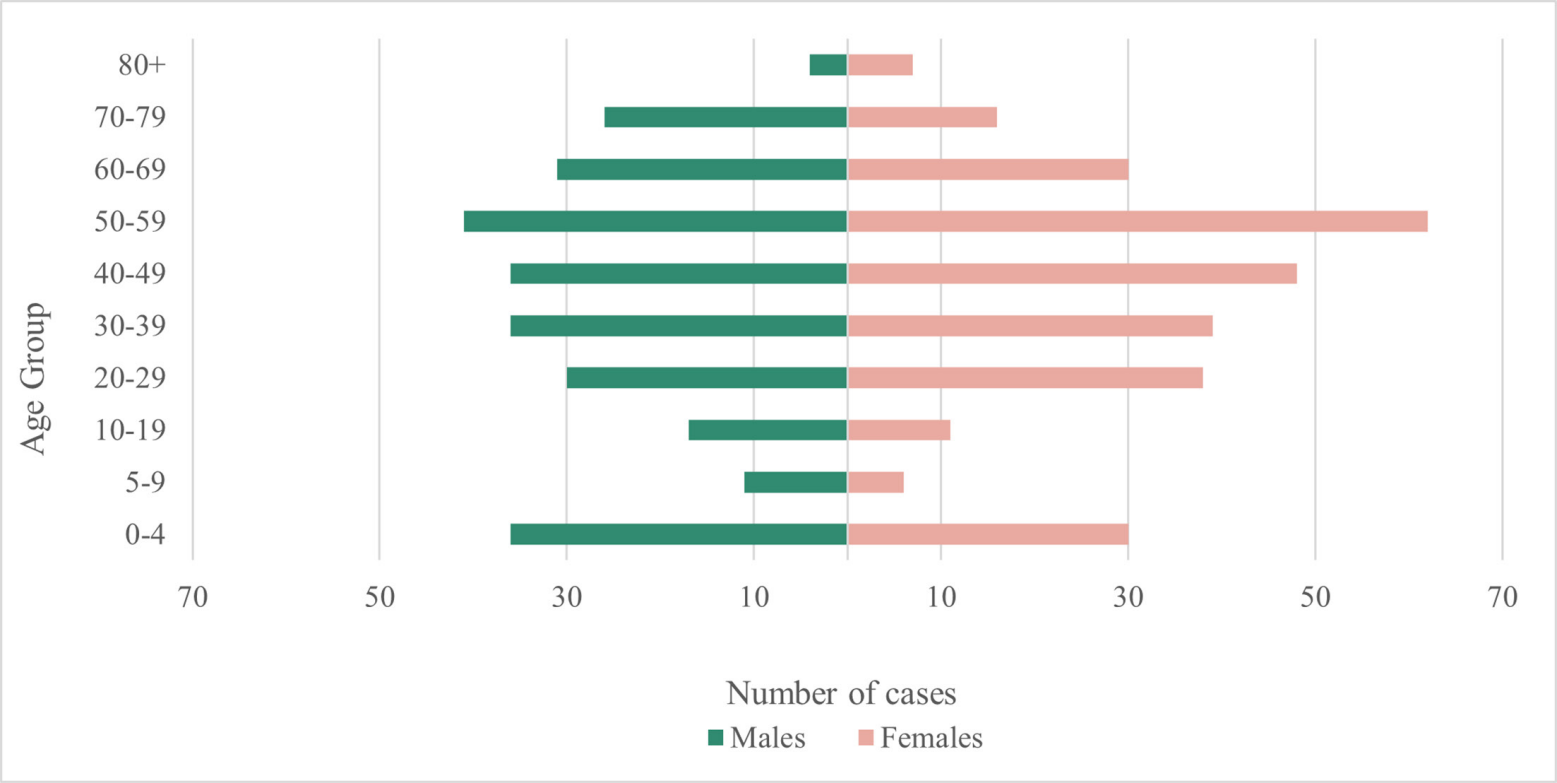
Age–sex distribution of total ETEC cases reported to UKHSA in England, where date of birth, sample receipt date and sex were available (*n*=555).

**Fig. 3.**
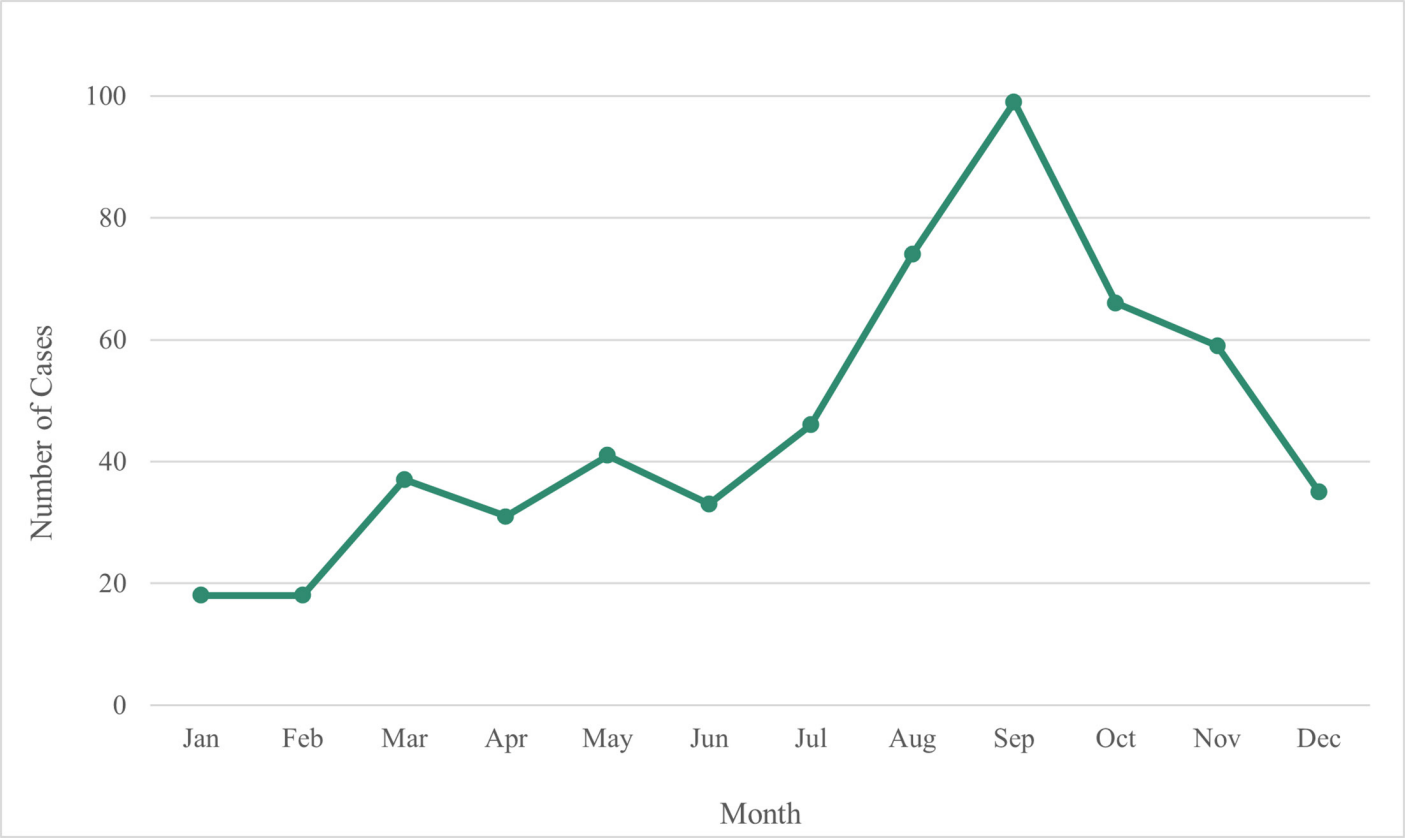
Seasonal variation in the number of ETEC cases: trends and patterns over a 9-year period (*n*=557).

**Fig. 4.**
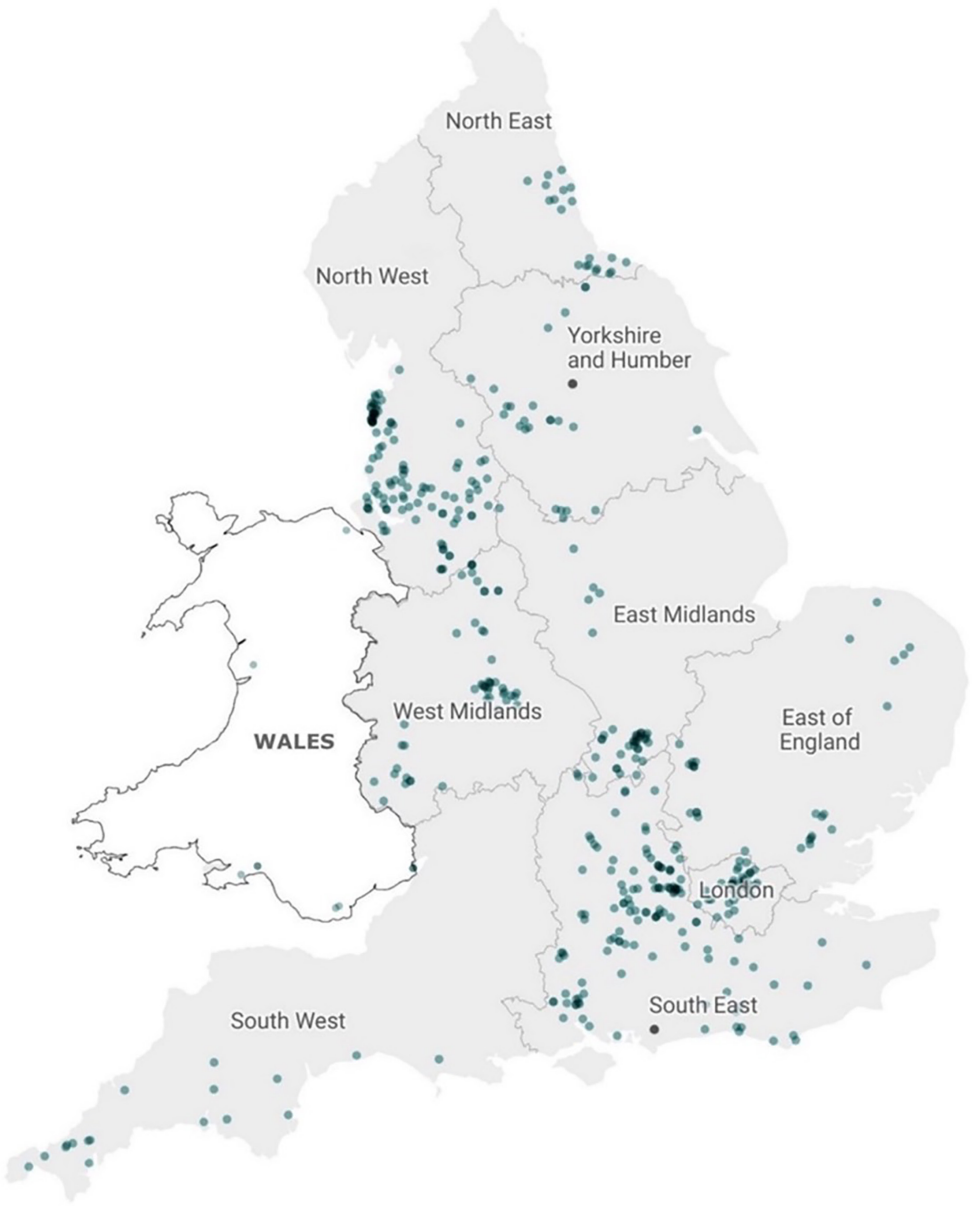
Geographical distribution of ETEC cases in England based on available patient postcodes (*n*=438/557). Note: seven cases had Welsh residential postcodes, but their samples were referred to laboratories in England for testing.

Age and sex data were available for 555 cases, and overall, the incidence was higher for females (51.7%, *n*=287) compared to males (48.3%, *n*=268). There was a high incidence of cases in the age group 0–4 (*n*=66/555); however, the highest incidence was observed in the age group 50–59 across both sexes (18.6%, *n*=103/555), with a higher frequency in female cases (60.2%, *n=*62) compared to male cases (39.8%, *n=*41). There was no notable difference in the sex distribution for all other age groups (Fig. 2). Seasonal variation was observed, with the lowest incidence in winter and a late summer/early autumn peak in September (Fig. 3). The geographical distribution of ETEC cases in England showed the highest incidence in the South East (*n=*116/438; 26.5%), North West (*n=*95/438; 21.7%) and London (*n=*74/438; 16.9%) (Table 1 and Fig. 4).

**Table 1.** Geographical distribution of ETEC cases by UKHSA Centre in England, where UKHSA centre information was available (*n*=438)

UKHSA Centre	Cases (%)
South East	116 (26.5)
North West	95 (21.7)
London	74 (16.9)
West Midlands	43 (9.8)
East of England	31 (7.1)
East Midlands	25 (5.7)
Yorkshire and Humber	21 (4.8)
South West	18 (4.1)
North East	15 (3.4)
**Total**	**438**

**Fig. 5.**
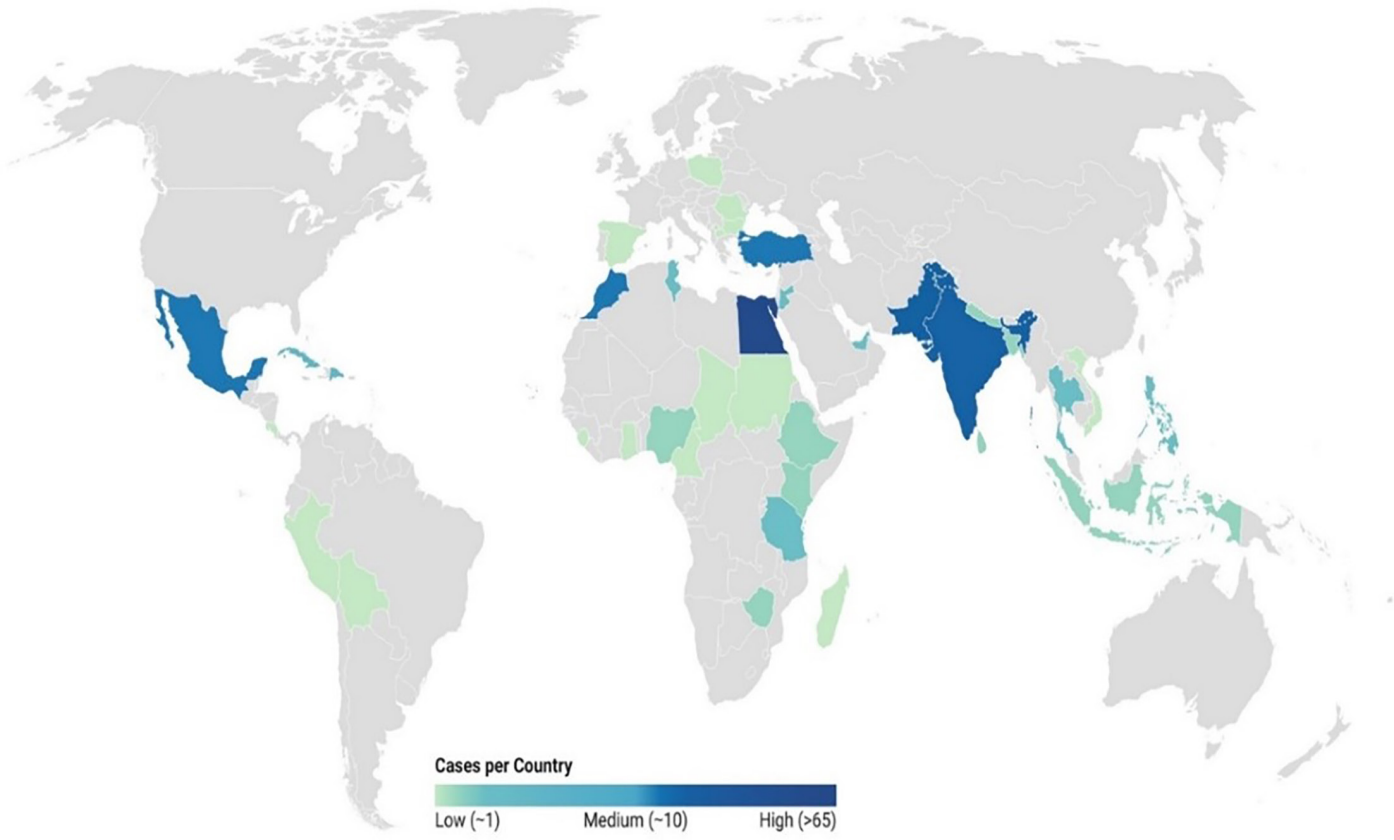
World map showing the incidence of ETEC cases based on available travel data (*n*=257). The gradient scale represents countries with lower case numbers in light colours, and darker colours were associated with a higher number of cases.

Travel history was reported for 49.5% (*n=*276) of cases, with 257 specifying at least one country and 19 not disclosing their destination. Unknown travel status was noted in 41.8% (*n*=233) of cases, while 8.6% (*n*=48) reported that they had not travelled outside the UK. The highest proportion of cases reported travel to Africa (21.5%, *n=*120/557), followed by Asia (16.9%, *n=*94/557) (Table 2), and the most frequently reported destinations were Egypt (12%, *n=*67/557), Pakistan (4.7%, *n=*26/557), India (4.5%, *n=*25/557), Turkey (2.7%, *n=*15/557) and Mexico (2.7%, *n*=15/557) (Fig. 5).

**Table 2.** Incidence of ETEC cases and their link to foreign travel, grouped by continent (*n*=557)

Travel information	Cases (%)
**Africa**	120 (21.5)
**Asia**	94 (16.9)
**North America**	30 (5.4)
**Europe**	8 (1.5)
**South America**	3 (0.5)
**Multiple continents**	2 (0.4)
**Yes – not stated**	19 (3.4)
**No travel**	48 (8.6)
**Unknown**	233 (41.8)
**Total**	**557 (100)**

Genomic MLST profiling identified 139 STs, with the most prevalent being ST182 (14.3%, *n*=84/587), followed by ST4 (12.8%, *n*=75/587) (Table S1). Genome-derived serotyping detected 132 serotypes, with O6:H16 (19.8%, *n*=116/587) and O169:H41 (11.2%, *n*=66/587) being the most common (Table S2). The dominant ST-serotype profiles were ST4 O6:H16 (*n*=74), ST182 O169:H41 (*n*=66), ST316 O27:H7 (*n*=32) and ST2353 O6:H16 (*n*=27) (Fig. S1). Genome-derived virulence typing identified the presence of the genes *STp*, *STh* and/or *LT* either individually or in combination. *STp* was the most frequently observed (*n*=180/587), followed by *STh* (*n*=127/587), *LT/STh* (*n*=114/587), *LT* (*n*=107/587) and *LT/STp* (*n*=58/587) (Fig. S2).

**Fig. 6.**
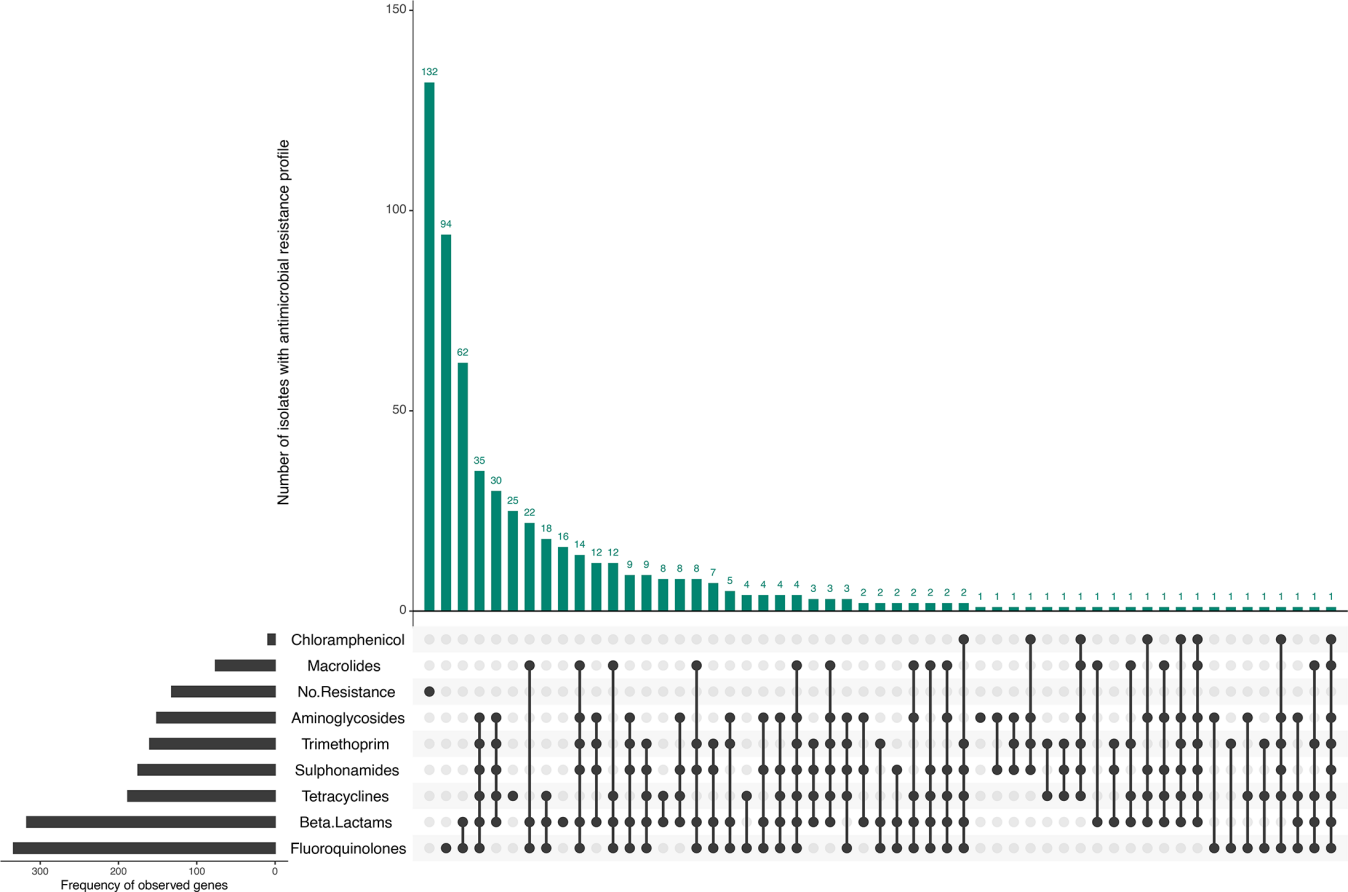
AMR profiles of ETEC isolates (*n*=587). Vertical bars represent the number of isolates carrying one or a combination of AMR genes categorized by antimicrobial class. Horizontal bars represent the frequency of observed AMR genes categorized by antimicrobial class. The figure was generated using UpSetR in RStudio.

Genome-derived AMR typing data were available for all isolates (*n=*587), of which 22.49% (*n=*132) harboured no AMR determinants from the reference database, and complete susceptibility to all eight antimicrobial classes was inferred. However, 77.51% (*n=*455/587) were observed to harbour AMR determinants known to confer resistance to at least one antimicrobial class (Fig. 6). Fluoroquinolone resistance was the most frequently observed profile (*n*=94), followed by fluoroquinolone and *β*-lactam resistance (*n*=62) (Figs 6 and S3). Isolates exhibiting resistance to three or more different antimicrobial classes were categorized as MDR. In the study population, 240 isolates were classified as MDR (Fig. 6 and Table S3).

*β-Lactams*. Out of 587 isolates, 53% (*n=*311) harboured AMR determinants known to confer resistance to the *β*-lactams. The most prevalent genes detected encoded the extended-spectrum *β*-lactamase (ESBL) *bla_CTX-M-15_* (*n=*148) or the penicillinase *bla_TEM-1_* (*n=*183) (Table S3).

*Aminoglycosides*. AMR determinants known to confer resistance to aminoglycosides were detected in 45.5% (*n=*267/587) of the isolates. Of these, *strA [aph(3’)-Ib]* (*n=*108) and *strB [aph(6’)-Id]* (*n=*98) were the most prevalent (Fig. 6 and Table S3).

*Fluoroquinolones*. A total of 56.9% (*n=*334/587) isolates harboured AMR determinants known to confer resistance or reduced susceptibility to the fluoroquinolones. The most frequent mutation observed in the quinolone resistance-determining region of the *gyrA* gene occurred at position 83, where serine (S) was substituted to leucine (L) (*gyrA 83:S-L*, *n=*158). The most frequently detected plasmid-mediated quinolone resistance gene was *qnrS1* (*n=*143) (Fig. 6 and Table S3).

*Macrolides*. AMR determinants known to confer resistance to macrolides were observed in 13.3% (*n=*78/587) of the isolates, with the *mphA* gene being the most prevalent (*n=*73) (Fig. 6 and Table S3).

*Trimethoprim and sulphonamides*. A total of 28.4% (*n=*167/587) isolates were observed to harbour *dfrA* variants known to confer resistance to trimethoprim, with *dfrA8* being the most prevalent gene (*n=*54) (Fig. 6 and Table S3). Out of 587 isolates, 31.7% (*n*=186) isolates presented genes known to confer resistance to sulphonamide, with *sul2* being the most common (*n=*126) (Fig. 6 and Table S3).

*Tetracyclines and phenicols*. AMR determinants known to confer resistance to tetracyclines were found in 32.5% (*n=*191/587) of the isolates, with *tetA* being the most frequent (*n=*188) (Fig. 6 and Table S3). Resistance to the phenicols was the least prevalent (1.7%, *n=*10/587). There were only two AMR determinants conferring resistance to the phenicols, which were identified as *catA1* (*n*=5) and *floR* (*n*=2) (Fig. 6 and Table S3).

## Discussion

The increase in notifications of ETEC cases between 2015 and 2023 was due in part to the progressive implementation of GI PCR assays across England since 2013. The sharp decline in 2020 and 2021 was attributed to nationwide lockdown measures during the COVID-19 pandemic, including international travel restrictions, the promotion of strict hygiene practices and social distancing and reduced case ascertainment, as shifts in public health-seeking behaviours and disruptions to surveillance efforts affected case detection and reporting [27,28]. This trend mirrors patterns observed globally in European and Asian countries and other GI pathogens in England [29,31]. ETEC notifications rebounded after restrictions were lifted in 2022 and included a small outbreak of hybrid STEC-ETEC [32]. The increasing case numbers align with trends observed elsewhere and may be linked to increased travel, rising AMR within the pathotype and enhanced molecular detection methods that have improved case detection [33]. Foodborne outbreaks of ETEC have been linked to foods imported from ETEC-endemic regions, driving greater interest in the surveillance of DEC pathotypes beyond STEC, stricter food handling regulations and enhanced ETEC testing algorithms [34,36].

The majority of cases were among individuals aged between 20 and 59, most likely driven by a higher frequency of international travel in this age group [7,33]. In LMICs, the prevalence of ETEC among children is high, and we also observed a relatively high incidence in children under 5 years old [37,38]. ETEC cases were unevenly distributed across England, with the highest incidence recorded in the South West and North West, and dense clusters around metropolitan areas. This may be explained by a greater number of local frontline laboratories implementing PCR-based detection in these areas. Over recent years, this trend has been observed for other DEC pathotypes in England [31].

In line with other studies, ETEC incidence in England exhibited distinct seasonal patterns [39]. Elevated ETEC incidence has been reported in tropical and subtropical regions such as Pakistan, India and Bangladesh, especially during the monsoon season [40]. Increased international travel during summer months, particularly to ETEC-endemic regions, increases the chances of exposure to contaminated food and water. In regions such as Pakistan, the rainy season accelerates ETEC spread through faecal-contaminated water, which elevates the risk of travel-associated infections during the summer months [41].

Approximately 40% of the cases were missing recorded travel history. Incomplete travel information impacts the accuracy of travel-acquired ETEC infections in England and results in a gross underrepresentation of the role of foreign travel in ETEC transmission. As previously described, a high proportion of cases were associated with international travel to Egypt, Pakistan, India, Turkey and Mexico [39,42, 43]. ETEC have been detected in raw beef in Egypt, shellfish from India and poultry and vegetables in Turkey, providing evidence of the potential for foodborne transmission and the possibility of UK travellers acquiring ETEC infections after consuming contaminated food abroad [44,46]. This study also highlighted the extensive genomic diversity of ETEC, although the most common serogroup *E. coli* O6 was also the most frequently detected ETEC serogroup in studies in Korea, Japan, the USA and elsewhere [47,51].

Different toxin profiles appear to be more prevalent in different regions [52,53]. Both *STh* and *STp* variants were identified in this dataset, either individually or in combination, and have been associated with greater disease severity among children in LMICs, highlighting the need for a more detailed assessment of the presence of each toxin variant and of clinical outcome [54].

Notifications of MDR ETEC are on the rise in LMICs, including India and other South Asian countries, where inadequate hygiene practices, contaminated water sources and antibiotic misuse may facilitate the spread of AMR between environments, with travellers playing key roles in global AMR dissemination [17]. High levels of AMR, based on genome-derived *in silico* AMR profiles, mirrored trends seen in Bangladesh [55]. Varying resistance patterns were highlighted, with the most detected genes conferring resistance to *β*-lactams, aminoglycosides and fluoroquinolones. The exclusive use of aminoglycosides for ETEC infections in veterinary medicine likely facilitated the transfer of AMR genes from animals to humans, complicating treatment and accelerating the spread of resistance [56,57]. Fluoroquinolone-resistant ETEC strains have also been linked to travellers, possibly contributing to the acquisition of AMR in domestically acquired ETEC strains [58,60]. The rising prevalence of ESBL-producing ETEC globally, particularly among travellers, supports the idea of their role in the global transmission of AMR [17,59, 61,65].

Expanding the implementation of PCR-based diagnostic methods in frontline laboratories across England will enhance the microbiological detection of ETEC and other DECs. Precise recording of travel history will facilitate more accurate collection of epidemiological data. Understanding of the clinical and public health burden of ETEC depends upon integrating both microbiological and epidemiological data to provide a precise assessment of pathogenicity, exposures and transmission routes. Sharing of genomic data at a local, national and international level will be vital in guiding public health interventions and informing enhanced surveillance strategies. Future studies are in progress to better understand the role of ETEC colonization factors in infection and to compare phenotypic AMR with the genotypic data.

## Supplementary material

10.1099/jmm.0.002081 Uncited Supplementary Material 1.

10.1099/jmm.0.002081 Uncited Supplementary Material 2.

## Abbreviations

AMR: antimicrobial resistance
DEC: diarrhoeagenic *Escherichia coli*
ESBL: extended-spectrum *β*-lactamase
ETEC: enterotoxigenic *Escherichia coli*
GDW: Gastro Data Warehouse
GI: gastrointestinal
LMICs: low- and middle-income countries
NCBI: National Center for Biotechnology Information
SRST: short-read sequence typing
ST: sequence type
STEC: Shiga toxin-producing *Escherichia coli*
TD: traveller’s diarrhoea
UKHSA: UK Health Security Agency
