# Supplementary material for: Surveillance of genomic diversity and antimicrobial resistance in enterotoxigenic Escherichia coli in England, 2015–2023
Source: J Med Microbiol. 2025 Oct 3;74(10):002081. doi: 10.1099/jmm.0.002081 (PMC12501468; doi:10.1099/jmm.0.002081)
Supplement: Uncited Supplementary Material 1. [file jmm-74-02081-s001.pdf]

Supplementary Figures

Supplementary Figure 1: Minimum spanning tree using the Achtman 7-gene MLST showing all STs categorised based on their serotypes (n=585). Bubble size correlates to the number of isolates

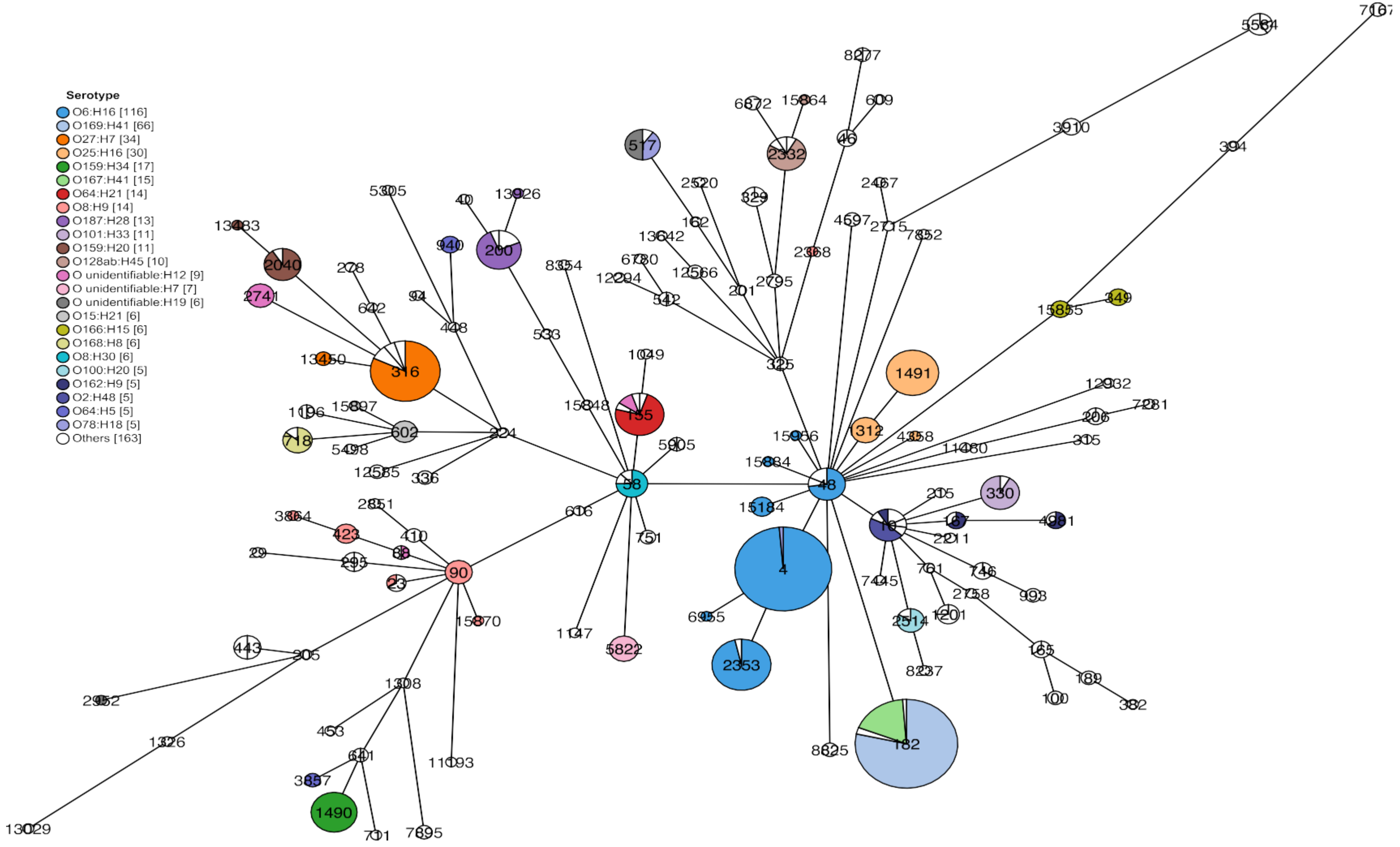

Supplementary figure 2*: Minimum spanning tree using the Achtman 7-gene MLST showing all STs categorised based on their virulence genes (n=586). Bubble size correlates to the number of isolates*

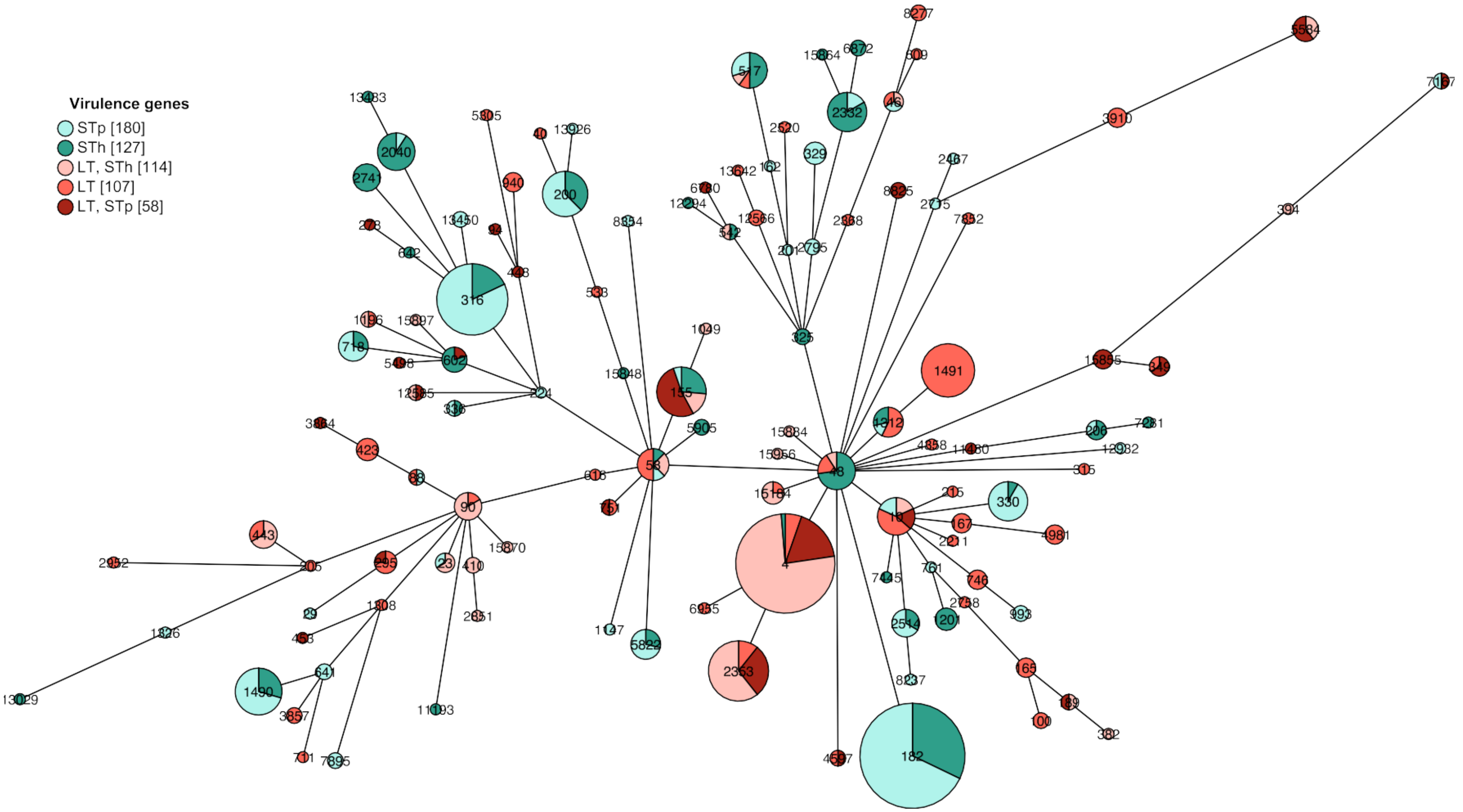

Supplementary figure 3: *Minimum spanning tree using the Achtman 7-gene MLST showing all STs categorised as susceptible, resistant, MDR, or XDR. Bubble size correlates to the number of isolates (n=586). Classification is based on the presence of genes known to confer resistance to antimicrobial classes: susceptible (0 classes), Resistant (defined as 1–2 classes), multidrug resistant (MDR defined as ≥3-5 classes), and extremely drug resistant (XDR define as >5 classes).*

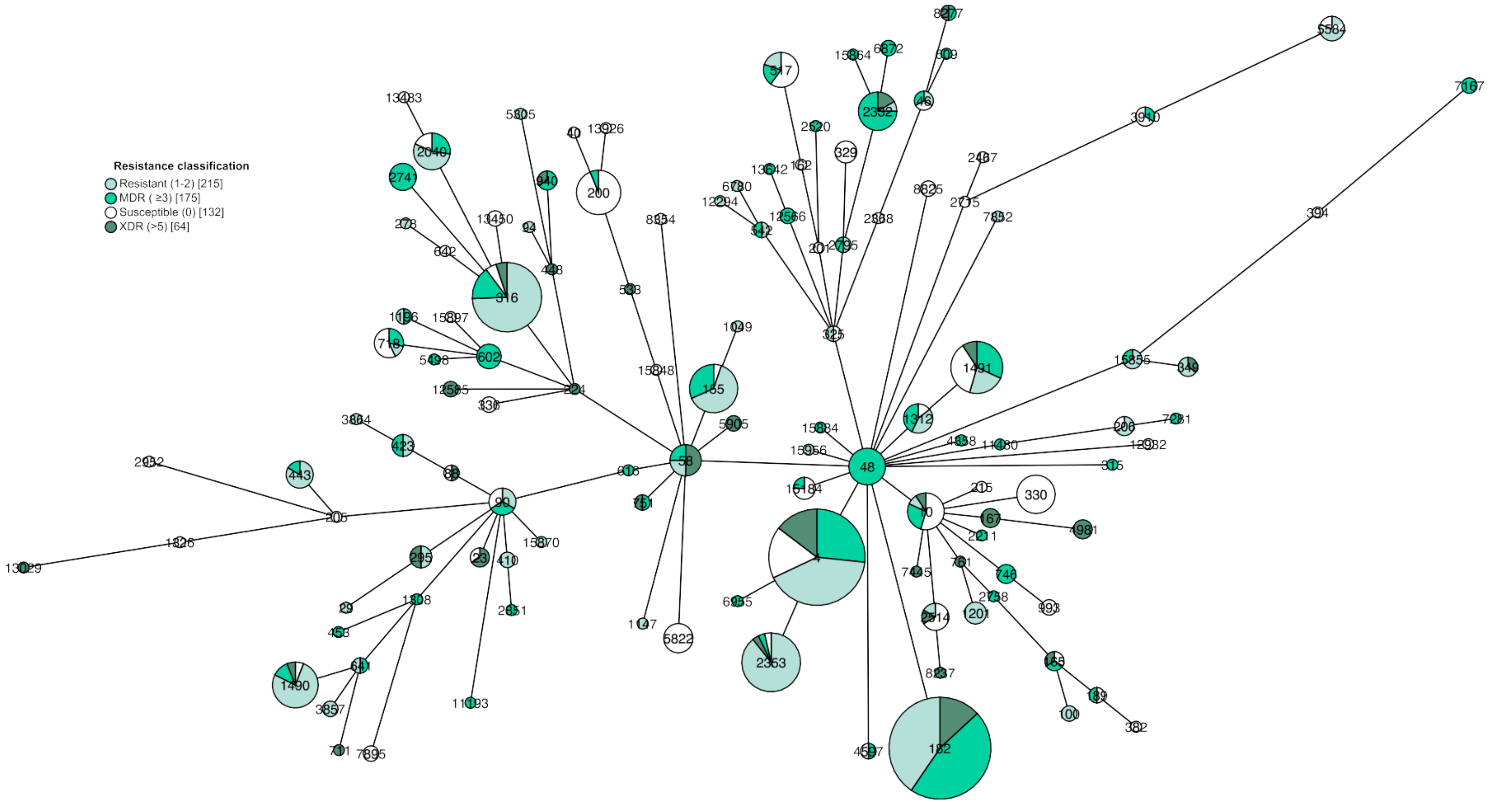

Supplementary Tables

Table 1*: Diversity of STs of ETEC within the study population (n=587). Other STs (96) - STs with less than 5 isolates. Failed* - The ST could not be assigned due to missing or insufficient sequenced data (incomplete locus coverage)*

ST	Cases (%)
182	84 (14.3)
4	75 (12.8)
316	39 (6.7)
2353	28 (4.8)
1491	22 (3.7)
155	19 (3.2)
1490	17 (2.9)
200	16 (2.7)
2332	12 (2)
330	12 (2)
2040	11 (1.9)
48	11 (1.9)
10	11 (1.9)
517	10 (1.7)
58	8 (1.4)
5822	7 (1.2)
1312	7 (1.2)
718	7 (1.2)
2741	6 (1)
2514	6 (1)
443	6 (1)
90	6 (1)
5584	5 (0.9)
602	5 (0.9)
Others (96)	156 (26.6)
Failed*	1 (0.1)
Total	587 (100)

Table 2*: Diversity of serotypes of ETEC within the study population. Serotypes found in less than 10 isolates were classified as "Others" (n=120).*

**Serotype**	**Cases (%)**
O6:H16	116 (19.8)
O169:H41	66 (11.2)
O27:H7	34 (5.8)
O25:H16	30 (5.1)
O159:H34	17 (2.8)
O167:H41	15 (2.6)
O8:H9	14 (2.4)
O64:H21	14 (2.4)
O187:H28	13 (2.2)
O101:H33	11 (1.9)
O159:H20	11 (1.9)
O128ab:H45	10 (1.7)
Others (120)	236 (40.2)
Total	587 (100)

Table 3: *Distribution of AMR genes, resistance phenotype and gene frequencies in the ETEC population*

Resistance Gene	Antimicrobial class	Resistance Phenotype	Count (%)
$bla_{CTX\text{-}M\text{-}12}$	β-lactams	AMO, AMP, AZT, CEP, CTA, CTZ, CTR, PIP, TIC	1 (0.06)
$bla_{CTX\text{-}M\text{-}15}$	β-lactams	AMO, AMP, AZT, CEP, CTA, CTZ, CTR, PIP, TIC	148 (9.12)
$bla_{CTX\text{-}M\text{-}65}$	β-lactams	AMO, AMP, AZT, CEP, CTA, CTZ, CTR, PIP, TIC	1 (0.06)
$bla_{DHA\text{-}1}$	β-lactams	AMO, AMC, AMP, AML, CTA, CXI, CTZ, PIP, PIT, TIC, TIL	8 (0.49)
bla_{mecA}	β-lactams	AMO, AMC, AMP, AML, CEP, CIX, CTA, CXI, CTZ, ERT, IMI, MER, PIP, PIT	1 (0.06)
$bla_{OXA\text{-}1}$	β-lactams	AMO, AMC, AMP, AML, CEP, PIP, PIT	3 (0.18)
$bla_{OXA\text{-}10}$	β-lactams	AMO, AMP, AZT, PIP, PIT	1 (0.06)
$bla_{SHV\text{-}12}$	β-lactams	AMO, AMP, AZT, CEP, CTA, CTZ, CTR, PIP, TIC	5 (0.31)
$bla_{TEM\text{-}1}$	β-lactams	AMO, AMP, CEH, PIP, TIC	183 (11.28)
$bla_{TEM\text{-}34}$	β-lactams	AMO, AMC, AMP, AML, PIP, PIT, TIC, TIL	1 (0.06)
$bla_{TEM\text{-}135}$	β-lactams	AMO, AMO, CEH, PIP, TIC	1 (0.06)
bla_{Z}	β-lactams	AMO, AMP, PCN, PIP	1 (0.06)
aac(3)-IVa	aminoglycosides	APR, GEN, DIB, NET, SIS, TOB	1 (0.06)
aadA1	aminoglycosides	SPE, STR	7 (0.43)
aadA2	aminoglycosides	SPE, STR	7 (0.43)
aadA5	aminoglycosides	SPE, STR	22 (1.36)
aadA8	aminoglycosides	SPE, STR	2 (0.12)
aadA15	aminoglycosides	SPE, STR	1 (0.06)
aadA17	aminoglycosides	SPE, STR	2 (0.12)
aadA23	aminoglycosides	SPE, STR	1 (0.06)
aadA24	aminoglycosides	SPE, STR	10 (0.62)
aadD	aminoglycosides	AMI, TOB	1 (0.06)
aph(3")-Ib	aminoglycosides	STR	1 (0.06)
aph(3')-IIa	aminoglycosides	BUT, KAN, NEO, PAR, RIB, GEN	1 (0.06)
aph(4)	aminoglycosides	HYG	1 (0.06)
aph(6)-Ic	aminoglycosides	STR	1 (0.06)
strA [aph(3'')-Ib]	aminoglycosides	STR	108 (6.65)
strB [aph(6)-Id]	aminoglycosides	STR	98 (6.04)
sat2A	aminoglycosides	STE	3 (0.18)
parC_EC2[56:A-T]	quinolones	CIP	3 (0.18)
parC_EC2[80:S-I]	quinolones	CIP	11 (0.68)
parC_EC2[80:S-R]	quinolones	CIP	1 (0.06)
parC_EC1[u]	quinolones	CIP	1 (0.06)
gyrA_EC2[82:S-L]	quinolones	CIP	12 (0.74)
gyrA_EC2[82:S-V]	quinolones	CIP	1 (0.06)
gyrA_EC2[83:S-A]	quinolones	CIP	6 (0.37)
gyrA_EC2[83:S-L]	quinolones	CIP	158 (9.74)
gyrA_EC2[87:D-B]	quinolones	CIP	1 (0.06)
gyrA_EC2[87:D-N]	quinolones	CIP	11 (0.68)

Resistance Gene	Antimicrobial class	Resistance Phenotype	Count (%)
gyrA_EC2[87:D-Y]	quinolones	CIP	2 (0.12)
gyrA_EC2[95:P-L]	quinolones	CIP	2 (0.12)
gyrA_EC2[u]	quinolones	CIP	7 (0.43)
qnrB2	quinolones	CIP	1 (0.06)
qnrB4	quinolones	CIP	8 (0.49)
qnrB19	quinolones	CIP	4 (0.25)
qnrS1	quinolones	CIP	143 (8.81)
ermB	macrolides	ERY, LIN, CLI, QUI, PIA, VIS	2 (0.12)
lnu(F)	macrolides	LIN	2 (0.12)
mef(B)	macrolides	ERY, AZI	1 (0.06)
mph(A)	macrolides	ERY, AZI, SPI, TEH	73 (4.50)
dfrA1	folate pathway antagonist	TRI, TRS	24 (1.48)
dfrA5	folate pathway antagonist	TRI, TRS	2 (0.12)
dfrA7	folate pathway antagonist	TRI, TRS	15 (0.92)
dfrA8	folate pathway antagonist	TRI, TRS	54 (3.33)
dfrA12	folate pathway antagonist	TRI, TRS	4 (0.25)
dfrA14	folate pathway antagonist	TRI, TRS	29 (1.79)
dfrA15	folate pathway antagonist	TRI, TRS	11 (0.68)
dfrA17	folate pathway antagonist	TRI, TRS	28 (1.73)
sul1	folate pathway antagonist	SME, TRS	56 (3.45)
sul2	folate pathway antagonist	SME, TRS	126 (7.76)
sul3	folate pathway antagonist	SME, TRS	4 (0.25)
tet(A)	tetracyclines	DOX, TET	188 (11.58)
tet(D)	tetracyclines	DOX, TET	1 (0.06)
tet(M)	tetracyclines	DOX, TET, MIN	1 (0.06)
tet(O)	tetracyclines	DOX, TET, MIN	1 (0.06)
catA1	phenicols	CHL	5 (0.31)
floR	phenicols	CHL	2 (0.12)

Note: *AMI, Amikacin; AMO, Amoxicillin; AMC, Amoxicillin+Clavulanic acid; AMP, Ampicillin; AML, Ampicillin+Clavulanic acid; APR, Apramycin; AZI, Azithromycin; AZT, Aztreonam; BUT, Butiromycin; CEP, Cefepime; CIX, Cefixime; CTA, Cefotaxime; CXI, Cefoxitin; CTZ, Ceftazidime; CTR, Ceftriaxone; CEH, Cephalothin; CHL, Chloramphenicol; CIP, Ciprofloxacin; CLI, Clindamycin; DIB, Dibekacin; DOX, Doxycycline; ERT, Ertapenem; ERY, Erythromycin; GEN, Gentamicin; HYG, Hygromycin; IMI, Imipenem; KAN, Kanamycin; LIN, Lincomycin; MER, Meropenem; MIN, Minocycline; NEO, Neomycin; NET, Netilmicin; PAR, Paromomycin; PIP, Piperacillin; PIT, Piperacillin+Tazobactam; PIA, Pristinamycin IA; QUI, Quinupristin; RIB, Ribostamycin; SIS, Sisomicin; SPE, Spectinomycin; SPI, Spiramycin; STR, Streptomycin; STE, Streptothricin; SME, Sulfamethoxazole; TEH, Telithromycin; TET, Tetracycline; TIC, Ticarcillin; TIL, Ticarcillin+Clavulanic acid; TOB, Tobramycin; TRI, Trimethoprim; TRS, Trimethoprim-Sulfamethoxazole; VIS, Virginiamycin S*
